# Assessing clinical decision support system tools in precision oncology: piloting ring testing

**DOI:** 10.1016/j.esmorw.2026.100731

**Published:** 2026-07-13

**Authors:** V. Nygaard, S. Zhao, D. Tamborero, R. Rosenquist, A. Stenzinger, P. Roepman, J. Lehtiö, C.W. Yde, K. Rekker, C. Brodin, L. Verlingue, D. Bordin, J. Klajic, S.F. Haj Mohammad, L. Silwal-Pandit, A. Nilsen, M.R. Teixeira, A. Peixoto, S. Nakken, E. Hovig, A. Edsjö, H.G. Russnes, PCM4EU Consortium

**Affiliations:** 1 Department of Pathology, Division of Laboratory Medicine, Oslo University Hospital, Oslo, Norway; 2 Department of Oncology-Pathology, Karolinska Institutet, Science for Life Laboratory, Stockholm, Sweden; 3 Division of Pathology, Karolinska University Hospital, Stockholm, Sweden; 4 Department of Molecular Medicine and Surgery, Karolinska Institutet, Stockholm, Sweden; 5 Clinical Genetics and Genomics, Karolinska University Hospital, Stockholm, Sweden; 6 Institute for Pathology, Heidelberg University Hospital, Heidelberg, Germany; 7 Hartwig Medical Foundation, Amsterdam, the Netherlands; 8 Center for Genomic Medicine, Rigshospitalet, Copenhagen, Denmark; 9 Department of Laboratory Genetics, Genetics and Personalized Medicine Clinic, Tartu University Hospital, Tartu, Estonia; 10 Centre de Recherche en Cancérologie de Lyon (CRCL), Centre Léon Bérard, Lyon, France; 11 Department of Clinical Molecular Biology, Akershus University Hospital, Unit for Precision Medicine, Lørenskog, Norway; 12 Department of Medical Oncology, Leiden University Medical Centre, Leiden, the Netherlands; 13 Department of Laboratory Genetics, Portuguese Institute of Oncology of Porto (IPO-Porto), Porto, Portugal; 14 Porto Comprehensive Cancer Center, Porto, Portugal; 15 School of Medicine and Biomedical Sciences (ICBAS), University of Porto, Porto, Portugal; 16 Department of Tumor Biology, Institute for Cancer Research, Oslo University Hospital, Oslo, Norway; 17 Center for Cancer Cell Reprogramming, Institute of Clinical Medicine, University of Oslo, Oslo, Norway; 18 Department of Clinical Genetics, Pathology and Molecular Diagnostics, Skåne University Hospital, Lund, Sweden; 19 Division of Pathology, Department of Clinical Sciences, Lund University, Lund, Sweden; 20 Department of Cancer Genetics, Institute for Cancer Research, Division of Cancer, Oslo University Hospital, Oslo, Norway; 21 Institut for Clinical Medicine, University of Oslo, Oslo, Norway

**Keywords:** clinical decision support, comprehensive genomic analyses, molecular pathology, precision oncology, quality assessments, ring test

## Abstract

**Background:**

The EU4Health project PCM4EU aimed to improve survival rates and quality of life of patients with cancer based on precision cancer medicine. To achieve this, enhanced expertise and quality of molecular cancer diagnostics are key. Clinical decision support systems (CDSS) have become increasingly important after the introduction of comprehensive genomic diagnostic profiling. While external quality assessment schemes are mandatory for most diagnostic laboratory tests, similar programs for CDSS tools are currently lacking. To address this, we piloted an international ring test documenting CDSS usage, performance, and manual interpretation.

**Materials and methods:**

Twenty synthetic datasets were generated, mimicking small variant call sets from a typical targeted 500-gene panel (VCF format) across multiple cancer types (10 tumour-normal pairs and 10 tumour-only). Participants received standardised instructions via e-mail and at a virtual meeting and submitted results using a structured response form.

**Results:**

Eight laboratories from seven countries participated. All participants submitted results for the 10 tumour-only cases; one submitted results from two assessors. Tumour-only cases contained 6-18 variants where interpretation could be critical. Oncogenic calls for hotspot variants showed good agreement across the various CDSS tools applied; however, variability existed regarding reported variants and clinical interpretation.

**Conclusion:**

The pilot ring test revealed clinically relevant discrepancies between laboratories and interpreters, underscoring the need for structured external quality assessment schemes for CDSS tools in addition to the existing laboratory workflow schemes. It also highlighted several challenges related to the generation of realistic synthetic data, the design of reporting formats, the definition of ground truth, and the manual interpretation of results.

## Highlights

• CDSS tools and manual interpretation are used for reporting tumour sequencing data.
• A well-designed and structured ring test is needed to secure the quality of testing.
• Ring tests should include CDSS comparison and evaluate manual curation.
• Ring tests with a few cases conducted regularly can ensure quality and safety.

## Introduction

Genetic testing strategies for personalised cancer medicine (PCM) have rapidly moved towards comprehensive genomic profiling (CGP), including large cancer gene panels as well as whole-exome or whole-genome sequencing.^1^ Although the primary use for diagnostic assays is detection of alterations relevant for routine therapy decisions, it can identify patients who may benefit from non-standard-of-care options, including biomarker-driven oncology trials.^2^ Clinical trials designed for drug repurposing (e.g. DRUP^3^, ProTarget^4^, and IMPRESS-Norway^5^) include patients with rare and often less well-defined variants in cancer-related genes, requiring substantially more comprehensive variant interpretation. Furthermore, the rapid increase in new classes of targeted therapies and the number of CGP analyses carried out have made data analysis and interpretation increasingly labour-intensive.

Data output from CGP assays undergoes analyses that typically include (i) preprocessing, alignment, and variant calling; (ii) variant annotation, including proposed functional impact on the protein; and (iii) evaluation of preclinical and clinical evidence supporting a variant’s clinical implication.^2^ Step 1 is often software provided by assay vendors, while steps 2 and 3 generally need to be carried out by the laboratories. Some use off-the-shelf solutions, while others have in-house developed tools. In both cases, there is a need to integrate data with external knowledge sources and to incorporate some level of manual curation and interpretation. Most laboratories have access to a molecular tumour board (MTB) facilitating cross-disciplinary review of results and providing treatment recommendations.^6^ The quality of CDSS tools and the clinical interpretation is therefore crucial for informed MTB discussions and/or appropriate reporting.

A range of CDSS tools is available, and the main objectives focus on steps 2 and 3 above, thereby assigning a level of variant actionability based on curated evidence [e.g. an ESCAT level (European Scale for Clinical Actionability of molecular Targets^7^) or other tier-based systems^8^]. The highest level of actionability is tier1/ESCAT1, corresponding to variants that are validated targets of a drug applied in standard of care for the patient’s tumour type. It is essential to optimise, standardise, and harmonise such processes to ensure Good Laboratory Practice and equitable access to molecular-driven cancer care. However, recent reports have indicated discrepancies in the interpretation of CGP assays and subsequent treatment decisions.^9^^,^^10^ In accordance with EU regulations for *in vitro* diagnostics regulation and medical devices regulation, robustness and accuracy of such tools must be continuously monitored to ensure patient safety.^11^ There is therefore a critical need to develop programs for quality assessments and to establish benchmarking procedures.

The PCM4EU project was launched in January 2023, involving partners from 15 European countries.^12^ As part of the project, we piloted a ring test for evaluation of CDSS tools by providing a synthetic dataset of CGP results designed to mimic patient data from selected tumour types. Participants processed the data through their standard pipeline for data interpretation, reported the tools used, and submitted their outputs in a structured form, specifically including the information they would have presented at an MTB meeting for each of the simulated cases.

## Material and methods

**Figure 1:**
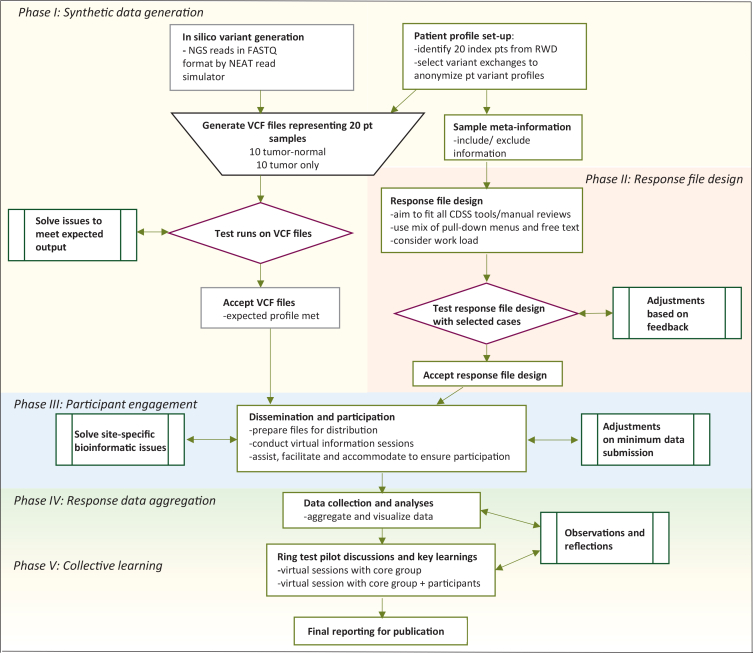
**Overview of the ring test workflow.** The ring test had five phases, where the first were the design and construction of the datasets. The second, design of the response form, was carried out in parallel. This was followed by a third phase where potential participants were contacted, briefed and engaged. The fourth phase was after the submission of results and consisted of data aggregation and analysis. The final, fifth phase was the reflection and discussion of learning points. CDSS, clinical decision support system; NGS, next-generation sequencing; RWD, real world data.

The core working group in WP2 in PCM4EU designed the ring test as a process with five phases, as shown in Figure 1. In the first phase, a total of 20 synthetic datasets were created mimicking both frequent and rarer molecular alterations found in certain tumour types (Supplementary File S1, available at https://doi.org/10.1016/j.esmorw.2026.100731). Ten of these datasets simulated results as if only tumour DNA had been analysed (Table 1), while the other 10 represented tumour-normal paired sequencing datasets (Supplementary Table S1, available at https://doi.org/10.1016/j.esmorw.2026.100731). For gene selection, the gene panel of the Illumina TSO500 DNA assay (TSO500; San Diego, CA, USA) was used as a template. In addition, synthetic germline data was created.

**Table 1:** Overview of the 10 synthetic patient cases representing tumour-only cases, including primary tumour type, subtype, and number of variants in the ‘true call set’

Sample ID	Primary tumour type	Tumour subtype	No. of variants in true call set
11	Colon/rectum	Sigmoid colon cancer, adenocarcinoma	11
12	Colon/rectum	Anal cancer, low-grade adenocarcinoma	12
13	Lung	Lung cancer, adenocarcinoma	18
14	Biliary tract	Cholangiocarcinoma	10
15	CNS/brain	Anaplastic astrocytoma	6
16	Head and neck	Salivary gland cancer	11
17	Breast	Breast cancer, triple negative	8
18	Breast	Breast cancer, lobular carcinoma	14
19	Kidney	Clear cell renal cell carcinoma	14
20	Neuroendocrine tumour	Pancreatic neuroendocrine tumour	10

ID, identification; CNS, central nervous system.

### Design of the response file

A response template in Excel file format was designed to capture both the variant annotation output of the CDSS tool and manual review (Supplementary File S2, available at https://doi.org/10.1016/j.esmorw.2026.100731). The response form contained (i) basic information regarding the ring test, (ii) a prefilled example response sheet with explanation, and (iii) a sheet requesting an overview of interpretation tools used, workflow, and competences involved. Twenty sheets were designated for the results of the cases. One section was used for reporting CDSS outputs, including classification of variants as oncogenic, likely oncogenic, variant of unknown significance (VUS), and drug(s) matched to variant. A second section was used for reporting manual curation/assessments and in a third section, the sites noted whether or not they would report the variant at their MTB. There was a mix of columns with pull-down menus and free text.

### Distribution, invitation, and participation

An invitation letter to participate was distributed by e-mail through the PCM4EU network on 7 April 2025 (Supplementary File S3, available at https://doi.org/10.1016/j.esmorw.2026.100731). Two virtual information sessions with Q&A sessions were held on 7 April 2025 and 11 April 2025. The Variant Call Format (VCF) files were distributed to the participants along with a read-me file and the response form. The 10 tumour-only sequencing datasets were required as mandatory (sample no. 11-20, Table 1), whereas the tumour-normal paired sequencing datasets were defined as optional (sample no 1-10, Supplementary Table S1, available at https://doi.org/10.1016/j.esmorw.2026.100731). After an extension of the deadline, participants were asked to respond by 2 May 2025 by submitting the response file, along with written comments on their experience.

## Results

### Workflow of the pilot ring test

As shown in Figure 1, the synthetic data sets were generated in phase I and internally tested to match the expected outcome. The constructed patient profiles all included oncogenic drivers that reflected ‘real-world’ tumour types, for example, a *KRAS* hotspot mutation in low-grade ovarian cancer (sample no. 1), *BRAF*, *APC*, and *SMAD4* mutations in colon cancer (sample no. 11) and a *VHL* mutation in kidney cancer (sample no. 19). For phase II, defining which parameters to report, phrases/nomenclature, as well as the design of the sheets itself was challenging, and a test run was carried out using one test case with prefilled somatic variants in the sample sheet. A second test run involved the distribution of a sample VCF file and revised response sheet to the core group for internal testing within their respective teams.

As most laboratories carried out tumour-only sequencing as standard, the tumour-normal pairs were labelled as optional. Engaging as many sites as possible, this reduced the number of mandatory samples required for participation from 20 to 10. The virtual information meetings were attended by network members from 11 countries. Nine response files were received, representing eight sites across seven countries (Supplementary Table S2, available at https://doi.org/10.1016/j.esmorw.2026.100731). Very few non-PCM4EU laboratories participated. The feedback from contacted sites revealed a too-tight time frame, challenged by planned routine diagnostic work. Two sites encountered difficulties loading VCF files into their pipelines, which were resolved by providing them with FASTQ files instead. For phase IV, all nine participants returned results for the 10 tumour-only mandatory samples, while only one site returned CDSS output for all 20 samples. One site submitted its own version of a response file, citing a lack of time to adhere to the original response form format. One participant did not use a CDSS tool, and hence, apart from the variants called, only the manual section was filled in the response sheets. Conversely, another participant left the manual section blank due to feasibility and time constraints. One site informed that their CDSS tool did not provide a tier classification of actionability, and thus they did not mark targetable variants in their reporting. Three sites reported using two CDSS tools (PCGR and MTB portal) due to complementary databases queried by the tools; for example, annotation from OncoKb is available through the MTB portal but is currently not included in the PCGR tool. In the sample sheets, columns with pull-down menus were more consistently filled than those with free text. Phase V was carried out as a virtual, collective session, where the results were discussed, but no individual feedback was given. The main feedback from participants involved technical feasibility to perform the ring test, shortcomings of the data and how to accommodate data in the response file with respect to the site’s own workflow and time constraints (Supplementary Table S3, available at https://doi.org/10.1016/j.esmorw.2026.100731).

### Concordance of CDSS output

**Figure 2:**
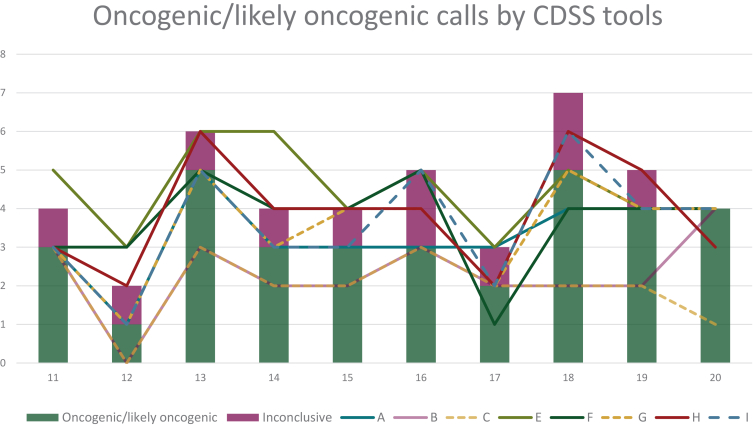
**Variants called oncogenic/likely oncogenic by CDSS tools.** The line series represents the total number of variants classified as oncogenic/likely oncogenic or inconclusive by the CDSS tools as reported per site. The stacked bars in the background show the expected total call. CDSS, Clinical decision support system.

The sites reported using a variety of CDSS tools as displayed in Table 2. All sites except two (E and G) utilised academic CDDS tools (publicly available). Three sites applied the same two tools (B, C, and I). For data analyses, variants functionally classified as oncogenic/likely oncogenic, either through CDSS-level annotation or manual review, were aggregated per site and per sample for cross-comparison of concordance/discrepancies and alignment with expected output (Table 3). From the CDSS reported data, we found that the number of variants classified as oncogenic/likely oncogenic by the CDSS tool at each site per sample was relatively similar (Figure 2). Sites B and C consistently reported fewer variants classified as oncogenic/likely oncogenic. The CDSS tool at site E called additional variants in three samples.

**Table 2:** Overview of the CDSS tools used by the participants

Site ID	CDSS tool	Specifications	Academic/commercial
A	MTB Portal (MTBP)	https://www.mtbp.org/	Academic
B	Personal Cancer Genome Reporter (PCGR), MTB Portal (MTBP)	https://github.com/sigven/pcgr https://www.mtbp.org/	Academic Academic
C	Personal Cancer Genome Reporter (PCGR), MTB Portal (MTBP)	https://github.com/sigven/pcgr https://www.mtbp.org/	Academic Academic
D	*None*		
E	VarSeq GoldenHelix	https://www.goldenhelix.com/	Commercial
F	CGI-Clinics	https://www.cgiclinics.eu/	Academic
G	Illumina Connected Insights	https://www.illumina.com/products/by-type/informatics-products/connected-insights.html	Commercial
H	Not reported		
I	Personal Cancer Genome Reporter (PCGR), MTB Portal (MTBP)	https://github.com/sigven/pcgr https://www.mtbp.org/	Academic Academic

Some of the commercial tools might have versions available for academic use.

CDSS, Clinical decision support systems; ID, identification.

**Table 3:** Overview of variants in each sample data set, which were expected to be classified as oncogenic/likely oncogenic and the reported numbers of variants (number of variants classified as oncogenic/likely oncogenic) reported from the CDSS tools per site (site D not included)

Sample no.	Expected variant classification		No. of CDSS oncogenic/likely oncogenic variant classified per site							
	Oncogenic/likely oncogenic	Inconclusive variant classification	A	B	C	E	F	G	H	I
11	BRAF:p.Asp594Gly APC:p.Glu1257Ter TP53:p.Gly266Glu	KRAS:p.Asp119His	3	3	3	5	3	3	3	3
12	TP53:p.Gln167HisfsTer4	PTEN:p.Leu112Gln	1	0	0	3	3	1	2	1
13	BRAF:p.Gly469Ala PIK3CA:p.His1047Arg TP53:p.Arg273Leu ARID2:p.Gln1100Ter BAP1:p.Ile32ArgfsTer41	PTEN:p.Leu112Gln	5	3	3	6	5	5	6	5
14	IDH1:p.Arg132Cys KRAS:p.Gly12Asp PBRM1:p.Arg710Ter	BAP1:p.Tyr173Cys	3	2	2	6	4	3	4	3
15	IDH1:p.Arg132His TP53:p.Arg273Ser ATRX:p.Val725GlyfsTer7	DNMT3A:p.Cys911Tyr	3	2	2	4	4	4	4	3
16	ERBB2:p.Val777Leu FBXW7:p.Arg465Cys HRAS:p.Gln61Arg	TP63:p.Arg594Ter PTEN:p.Leu112Gln	3	3	3	5	5	4	4	5
17	PIK3CA: p.Val105del PTEN: p.Gln87Ter	ERBB3:p.Arg103Gly	3	2	2	3	1	2	2	2
18	NF1:p.Gln400Ter MAP2K4:c.394-1G>A PIK3R1:p.Asp464_Tyr467del CDH1:p.Asp291AlafsTer6 GATA3:p.Leu130ProfsTer174	MAP3K1:p.Gln1494Ter DNMT3A:p.Cys911Tyr	4	2	2	5	4	5	6	6
19	VHL:p.Trp88Ter PBRM1:p.Phe379LeufsTer25 SETD2:p.Arg385AsnfsTer9 DNMT3A:p.Arg320Ter	DNMT3A:p.Cys911Tyr	4	2	2	4	4	4	5	4
20	TP53:p.Arg213Ter MEN1:c.1049+2T>G TSC2:p.Gln1380Ter CTNNA1:p.Gln678Ter	-	4	4	1	4	4	4	3	4

CDSS, clinical decision support system.

**Figure 3:**
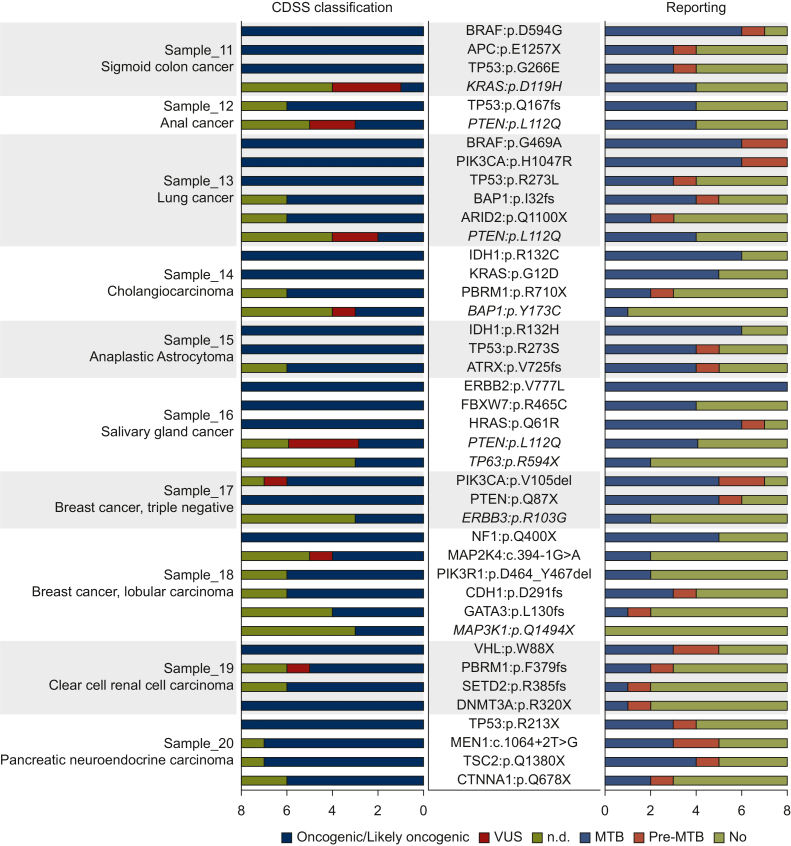
**Comparison of results pre- and postmanual curation of the CDSS outputs.** The variants with expected output as oncogenic/likely oncogenic per sample are listed in the middle, CDSS classification output to the left and whether the variant was reported at the MTB or pre-MTB on the right. The total length of the bar axis for both sides is 8, representing the eight sites that provided data for the respective sections. There are 40 gene variants listed, 33 that were expected to be classified as oncogenic/likely oncogenic and six variants (italic) that had inconclusive status (excluding *DNMT3A*:p.C911Y). CDSS*,* Clinical decision support system; MTB, molecular tumour board; VUS, variant of unknown significance.

Across the 10 samples, a total of 33 variants were expected to be annotated as oncogenic/likely oncogenic. CDSS tools showed complete agreement for 18 of these variants (Figure 3). In addition, 20 expected variants were classified as oncogenic/likely oncogenic by at least seven sites, and 30 variants by six or more sites. For the six preselected inconclusive variants (excluding *DNMT3A*:p.C911Y), classification as oncogenic/likely oncogenic by CDSS tools was reported by only two to three sites; for example, *ERBB3*:p.R103G was annotated as oncogenic at three sites.

Among the patient sample no. 11-20, only no. 14 was generated to have a level 1 biomarker (*IDH1*:p.Arg132Cys) in cholangiocarcinoma. From the response data, all but one site assigned this variant to this evidence class. The drug match in all responses was ivosidenib. However, the collected data also revealed unexpected tier1/ESCAT1 assignments for several variants. This may reflect that CDSS tools curate evidence not only at the individual variant level but also by mutation class and gene level. This issue is illustrated by the activating *ERBB2*:p.V777L mutation in patient sample no. 16 (salivary gland cancer), for which reported levels of evidence varied widely, ranging from level 1 to level 4. These findings highlight the need to contextualise, adjust, or filter the candidate actionable variants flagged by the CDSS tool.

### Manual variant review

We assessed the manual review of each tumour-only sample, that is, the reported manual variant interpretation. A few of the inconclusive variants switched functional class by manual review; for example, two sites interpreted the *KRAS*:p.D119H to be likely oncogenic, while the CDSS reported VUS. In contrast, one site interpreted the variant as a VUS, while the CDSS output stated likely oncogenic. We found six unexpected variants with oncogenic/likely oncogenic class identified by the CDSS that were all manually interpreted to be VUS. Two of these were missense mutations in clinically relevant genes (*NTRK1* and *ALK*).

From the manual review, we quantified the variants reported to be discussed at the MTB or pre-MTB (Figure 3). The bar plots, left versus right, show that many of the oncogenic/likely oncogenic variants were assessed not to be relevant for discussion at the MTB. Likely reasons are variants with no drug match, for example, tumour suppressors with unknown biallelic inactivation status, or site-specific reasons such as drug approvals/availability or no trial options. Consensus was only reached for one variant: *ERBB2*:p.V777L in a salivary gland cancer. The complexity of measuring concordance among participants concerning variants selected for MTB discussion is illustrated by sample no. 11 (Supplementary Table S2, available at https://doi.org/10.1016/j.esmorw.2026.100731). This case involved a colorectal tumour harbouring a *BRAF* class III mutation with additional driver mutations in *APC* and *TP53*. The presence of a *KRAS* mutation further complicated interpretation due to limited functional evidence available for that specific variant (*KRAS*:p.Asp119His). From the concatenated CDSS results across participants, the number of oncogenic/likely oncogenic variants identified in this sample ranged from three to five, with the participants agreeing upon classification as oncogenic/likely oncogenic for three of them (*BRAF*, *APC,* and *TP53*). All CDSS tools annotated the *BRAF* variant as oncogenic and MEKi, anti-EGFR, BRAFi, and chemotherapy drug classes were suggested as therapeutic matches to the variant. Five sites manually reported via free text that the variant was a class III *BRAF* mutation, while one site reported it as a class II, which could impact the clinical discussion. Furthermore, the *KRAS* variant was challenging: four sites did not report any annotation of the *KRAS* variant from their CDSS tool at one site, the variant was annotated as oncogenic, while for the remaining three sites, the variant was annotated as a VUS. However, after manual curation, two sites changed the CDSS annotation ‘VUS’ to likely oncogenic and clinically relevant as potential resistance to anti-EGFR therapy. For some MTBs, trial options for *BRAF* class III mutated CRC’s with an oncogenic/likely oncogenic *KRAS* variant might vary, and whether or not the *KRAS* variant would be identified as clinically relevant and reported at the MTB would thus differ.

## Discussion

Molecular pathology, and in particular DNA and RNA sequencing, has become a cornerstone of modern oncology and the use of large next-generation gene panel sequencing is rapidly increasing. The ongoing development in the field of PCM depends on the identification of patients with molecular alterations outside the traditional targets.^13^ This process is mainly driven by off-label (basket) trials, compassionate use programs, as well as early and late-phase clinical trials for new precision cancer medicines. Regardless of the indication for testing, laboratories must secure high quality and accuracy. Laboratory accreditation (specified by the International Standardization Organization, ISO) is associated with fewer analysis errors.^14^ In the initial ‘Next-Generation Sequencing Solid Tumour Proficiency Testing Survey’ by the College of American Pathologists, laboratories demonstrated a very high accuracy with regard to the detection of somatic single-nucleotide variants.^15^ One important tool to secure quality is participation in external quality assurance (EQA) programs, but interpretation and matching to clinical trials are generally not a part of the assessment.^16^ EQA providers are organisations designing and coordinating the schemes, in contrast to ring studies, which are often organised internally between laboratories.^17^ In accordance with EU regulations, validation of the analysis is obligatory, and participation in interlaboratory comparisons such as ring tests is important. Requirements and recommendations for the design of EQA or ring tests are defined by ISO^18^ and some main subjects are important.^16^ This is reflected in our ring test design, and we found each phase to have distinct challenges. Firstly, creating synthetic variant data sets mimicking real-world patients with cancer was suboptimal as the data did not simulate different allele frequencies and copy number variation, necessary for assessment of clonality, ploidy and purity, and calculation of complex biomarkers. Also, accompanying clinical information needs to be more detailed than what was provided in this ring test. Secondly, designing a report form to collect both CDSS output and manual interpretations was difficult to standardise. By using drop-down menus, we managed to standardise some of the response categories. Still, some laboratories found it challenging and laborious to fill in. The definition of the parameters to be reported could have been better clarified. As one participant commented: ‘In relation to the field “Manual review: current clinical relevance of variant at your institution”, it was not entirely clear how the term “variant of potential interest” should be interpreted. Is it in relation to treatment, tumour growth, signalling pathways activation, etc.? We ended up using the term widely’. This touches upon one of the main challenges in testing the quality of interpretation of gene variants in a clinical setting, defining the ground truth.

CDSS tools are under continuous development and rely on databases that are regularly updated. In order to assess an individual CDSS, care must be taken with the versions used, and validation must be carried out regularly. Further, compiling and comparing results was challenging, in particular as there is variability in access to treatment and trials, so what to report at MTB is highly variable. Even sites using the same CDSS tools differ in their manual review responses and MTB reporting. It might be that the definitions of the reporting categories in the forms were perceived differently by the participants. The results showed that manual curation and judgement are a key component, and better tailoring of a report form and providing individual feedback is of major importance. Ring tests represent arenas for learning and competence building and it is recommended to take part in such programs already when starting to implement a new test in the laboratory.^19^ Ring tests assessing interpretations and reporting represent a shift towards assessment of individuals, not only laboratories. Interestingly, two assessors from the same laboratory participated (B and C in Figure 2) and reported differently for one case. In addition, rapid advancements in machine learning and artificial intelligence are expected to improve CDSS tools.^9^ These systems are, however, not yet mature enough to support clinical diagnostics independently and manual curation by experts in the interpretation of molecular data remains essential. As not all cases should be discussed at an MTB, standardisation of reporting forms is important to avoid misunderstandings.^20^

Finally, the schedule was found too challenging for several laboratories as it competed with routine diagnostic work. This was in particular seen for non-PCM4EU partners, as they did not have the information upfront that a ring test was planned for the spring of 2025. However, if introduced as compulsory EQA schemes with time plans announced well in advance, turnaround times close to those of clinical diagnostics might still be feasible.

### Conclusion

Overall, our pilot identified several challenges that need to be addressed for a proper quality assessment scheme, including logistics, data design, compilation of cases, technical issues, response format, evaluation of results, and strategies to alleviate the workload, so as not to discourage sites from participating. Laboratories use a wide diversity of CDSS tools combined with different levels of manual interpretation and curation. A ring test only assessing the output of CDSS tools will be easier to design than a test for the complete procedure, that is, including the manual interpretation. The manual curation is instrumental for the quality of the results and a ring test including this part is also important for educational purposes. Future ring tests could include fewer cases and be conducted at regular intervals to help ensure the quality and safety of molecular diagnostics within precision oncology.
