# Supplementary material for: Assessing clinical decision support system tools in precision oncology: piloting ring testing
Source: ESMO Real World Data Digit Oncol. 2026 Jul 13;13:100731. doi: 10.1016/j.esmorw.2026.100731 (PMC13382446; doi:10.1016/j.esmorw.2026.100731)
Supplement: Supplementary File 1 [file mmc1.docx]

**Supplementary File 1**

**Material and Methods**

***Design of synthetic DNA sequencing data***

A total of 20 datasets were created mimicing both frequent and more rare molecular alterations found in certain tumor types (Table 1). Ten of these datasets simulated results as if only tumor DNA had been analysed, while the other ten represented tumor-normal paired sequencing datasets. For gene selection, the gene panel of Illumina TSO500 DNA assay was used as the template.

***Synthetic germline data:*** To generate germline single nucleotide polymorphism (SNP) profiles, polymorphism sites from the call set of eight in-house samples were combined to a union set. A total of 1200-1400 variants were down-sampled randomly from the union set for each patient sample, representing a germline variant profile of TSO500 synthetic data for normal control.

***Synthetic tumor (somatic) data:*** The somatic variant profiles of 20 synthetic tumor samples were based on real world TSO500 data from 20 pre-selected patient cases sequenced at Oslo University Hospital. The profiles represented a range of cancer types and harboured somatic variants relevant to biomarker status, actionability and/or oncogenicity. In each sample, 1-5 variants had been functionally classified by a CDSS tool as oncogenic/likely oncogenic (Table 1 and Supplementary Table 1). In addition, a few variants of inconclusive oncogenicity status were added to selected cases in order to investigate alignment across CDSS tools and subsequent manual interpretation of challenging variants. The sample profiles were anonymized by; 1) removing likely private germline variants, and 2) replacing selected functional variants with equivalent alternatives. In addition, one hypermutated sample with a high TMB was simulated by inserting 61 mutations. The average number of somatic variants (true call set) excluding the hypermutated sample, was 9 (range, 4-18). The pre-defined somatic variants, together with the germline variant profile, were used to generate synthetic reads representing tumor sample data. Tumor cell content was set to 100% and ploidy to 2.

***Generating synthetic data for 20 samples in fastq format***

The NEAT read simulator (version 3.0)^10^ was used to synthesize NGS reads data of the 20 samples. In the procedure of data generation in FASTQ format, key parameters (e.g., sequencing error statistics, read fragment length distribution and GC% coverage bias) were derived from the default data model provided by NEAT. Neither copy-number profile nor structural variants were introduced in the synthesis of tumor sequencing data due to a limitation of NEAT unable to model them in the current release. A few quality criteria were set for simulation: a) median read coverage in target region: 500-1000X for tumor sample and 400-800X for germline, b) read length: 94 bp for all datasets and c) insert size: 150bp ± 50bp. In addition, unique molecular identifiers (UMI) were added in the header of each read sequence of the synthetic data.

***Variant calling of the synthetic data via Illumina TSO500 LocalApp***

Variant calling on the synthetic read data from 20 samples was performed using Illumina TruSight Oncology 500 LocalApp (v2.1)^11^. Samples #01-10 were analysed in ‘tumor-normal pair’ mode, while samples #11-20 were analysed in ‘tumor-only’ mode.

The initial test run of the complete bioinformatics pipeline yielded an over-representation of pathogenic variants in the synthetic germline data. This was due to unfiltered selection of germline SNPs in RWD. Variants with VAF’s below the filter threshold (VAF<0.02) had been included, of which some were classified in ClinVar as pathogenic. The outcome thus yielded unrealistic germline profiles, which was resolved by removing these variants. A few of the pre-selected somatic variants were unintentionally inserted into several samples instead of unique samples, but due to time constraints, corrections were not performed.
