# Supplementary material for: Assessing clinical decision support system tools in precision oncology: piloting ring testing
Source: ESMO Real World Data Digit Oncol. 2026 Jul 13;13:100731. doi: 10.1016/j.esmorw.2026.100731 (PMC13382446; doi:10.1016/j.esmorw.2026.100731)
Supplement: Supplementary File 3 [file mmc3.pdf]

Invitation to participate in a ring-test for clinical decision support tools (CDSS)

The EU4health project PCM4EU aims at improving the survival rates and quality of life of cancer patients in the EU using PCM based on best practices. Data interpretation tools are important to reach this goal and within PCM4EU WP2 we have developed synthetic data for testing the performance of tools used for comprehensive genomic profiling analyses.

WHO can participate: the ring-test is open for all partners in the PCM4EU consortium and we anticipate that most will participate. Additionally, the ring-test is open for other laboratories that are interested in testing their routine practice. Feel free to forward the invitation. **RESPOND to vigdisny@ous-hf.no if you want to participate.**

WHAT will be provided:

- 20 datasets have been created (synthetic data): small variant call sets from synthetic TSO500 data in VCF format: 10 datasets have been generated to represent matching tumor and normal samples. The other 10 have been set-up in a tumor-only mode.
- One excel file which contains: Ring-test text explanation, a worksheet to fill in information concerning your CDSS tool(s), one worksheet for each of the 20 samples
- You do not need analyse all 20 cases to participate but results from at least the first ten cases (#1-#10) from each participating site is needed for the compilation of results.

HOW to perform the ring-test: We want to compare “standard” analytical workflows and ask that you use the pipeline and tools that are as close as possible to the everyday routine, including potential manual curation and interpretation. Please add information about the manual curation steps whenever applicable. We also ask for a brief overview of your workflow, the interpretation tools used and competences involved.

WHAT will the result be used for: The results from all participants will be compiled and analysed with a focus on which tools are used and how labs integrate the results from various tools and (manual) interpretation/curation; which variants seem to be more challenging to evaluate than others and whether or not the clinical impact will differ. The results will be discussed with all participants and published in a peer review paper.

WHAT are the limitations: the synthetic data cannot be used for copy number estimates or for variant allele fraction analyses. It means that for this ring-test study, gains/losses/amplifications will not be evaluated. Neither will bi-allelic events, subclonal alterations nor complex genomic biomarkers.

WHEN must the results be returned: The ring-test will close April 17th 2025.
