# Supplementary material for: Assessing clinical decision support system tools in precision oncology: piloting ring testing
Source: ESMO Real World Data Digit Oncol. 2026 Jul 13;13:100731. doi: 10.1016/j.esmorw.2026.100731 (PMC13382446; doi:10.1016/j.esmorw.2026.100731)
Supplement: Supplementary Table 1 [file mmc4.docx]

| Site | Topic | Comment |
| --- | --- | --- |
| G | VCF files and tertiary software input requirements | After consulting with the provider, it turns out that our bioinformatic pipeline is set up to start from FASTQ files only, so, unfortunately, we are not able to participate using a synthetic vcf file. This is because our tertiary software, Illumina Connected Insights, needs the fold and file structure that is automatically generated after processing the FASTQ files.    *Solution: The site was forwarded FASTQ files* |
| F | VCF files from normal samples and tertiary software input | I don’t think that CDSS tool accepts/identifies normal samples in VCF file |
| H | Disclaimer  attached with submission of response file | *Disclaimer*  1. A complete work-up of the cases is not possible because there is only information on nucleotide variants, whereas in real life one considers CNV, LOH, TMB, MSI and HRD. The data were discussed in our molecular tumor board setting with in mind the shortcomings of these data, thereby not leading to a valid advice.  2. Tumor suppressor genes can only be targeted when there is complete inactivation of both alleles. This cannot be assessed from the data given.  3. The panel we use is Oncomine plus, which is different from TSO500. A filter only including the Oncomine plus targets is used for the analysis. However the important diagnostic and druggable targets are present in our panel.  4. Classification is done differently, not with Tiers but with pathogenicity class, so we didn’t fill in your excel table but used our own format to be representative of our pipeline. Otherwise it would have taken too much time to transfer all results to your format. |
| E | Comments attached with submission of response file | We have some general feedback/comments about the setup and how we have reported relevant variants:    - All our results are manually transferred into the provided excel sheet incl. the automated output result which means that there might be errors related to copy/paste.  - Our CDSS tool is not set up to output the Tier classification (automated classification) so there might be discrepancies in this field as our output is not straight forward.  - We do not include treatment suggestions or mark mutations if targetable in the clinical genomic reports. This is discussed at weekly tumor board meetings with relevant clinicians. Therefore, we have marked “not manually assessed” in the “Manual review: variant actionability classification” and “Manual review: clinical significance/classification of variant as a resistance marker to therapy”. However, we have filled out “Manual review: current clinical relevance of variant at your institution” according to the knowledge about relevant variants/protocols from the national tumor boards.  In relation to the field “Manual review: current clinical relevance of variant at your institution”, it was not entirely clear who the term “variant of potential interest” should be interpreted. Is it in relation to treatment, tumor growth, signaling pathways activation etc. We ended up using the term widely |
| G | Comment that illustrates tumor-only as general set-up | We have analyzed the 10 cases of tumor-only TSO500 testing, as that is our usual setting. |

**Supplementary Table 3:** *Comments concerning the processes in the ring test provided by the participants.*
