# Supplementary material for: Assessing clinical decision support system tools in precision oncology: piloting ring testing
Source: ESMO Real World Data Digit Oncol. 2026 Jul 13;13:100731. doi: 10.1016/j.esmorw.2026.100731 (PMC13382446; doi:10.1016/j.esmorw.2026.100731)
Supplement: Supplementary Table 2 [file mmc5.docx]

| **Sample Id** | **Primary tumor type** | **Tumor subtype** | **# variants in true call set** |
| --- | --- | --- | --- |
| 1 | Ovary | LGSOC | 5 |
| 2 | Ovary | LGSOC | 5 |
| 3 | Ovary | Clear cell carcinoma | 10 |
| 4 | Ovary | Endometrial cancer, adenocarcinoma | 49 |
| 5 | Ovary | Poorly differentiated carcinoma of the ovary | 5 |
| 6 | Cervix | Cervix cancer, mucinous adenocarcinoma | 13 |
| 7 | Adrenal Gland | Adrenocortical carcinoma | 8 |
| 8 | Thyroid cancer | Anaplastic thyroid carcinoma | 7 |
| 9 | Bladder/Urinary Tract | Papillary urothelial carcinoma | 4 |
| 10 | Esophagus/Stomach | Esophageal adenocarcinoma | 12 |
