# Supplementary material for: Assessing clinical decision support system tools in precision oncology: piloting ring testing
Source: ESMO Real World Data Digit Oncol. 2026 Jul 13;13:100731. doi: 10.1016/j.esmorw.2026.100731 (PMC13382446; doi:10.1016/j.esmorw.2026.100731)

**Supplementary Table 2:** *Results sheets for responses to cases #11-20. See Supplementary file 2 for information about the categories.*

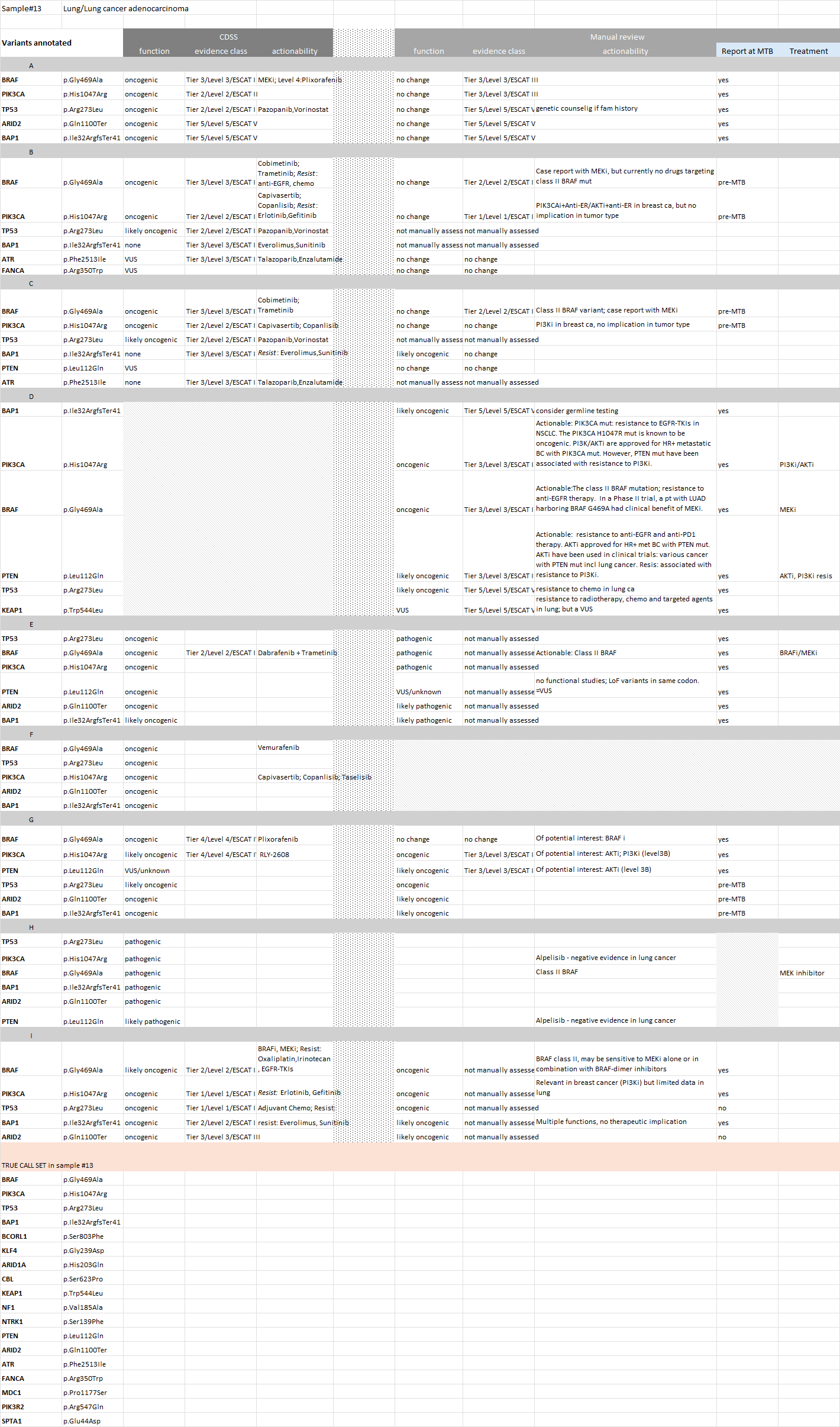

Supplement: Supplementary Table 3 [file mmc6.docx]
